# A Dose-Escalation Study Demonstrates the Safety and Tolerability of Cellobiose in Healthy Subjects

**DOI:** 10.3390/nu12010064

**Published:** 2019-12-25

**Authors:** Margret Irmgard Moré, Elisa Postrach, Gordana Bothe, Sonja Heinritz, Ralf Uebelhack

**Affiliations:** 1 analyze & realize GmbH, 13467 Berlin, Germany; mmore@a-r.com (M.I.M.); gbothe@a-r.com (G.B.); 2 Savanna Ingredients GmbH, 27404 Elsdorf, Germany; sonja.heinritz@savanna-ingredients.com (S.H.); 3 Charité-Universitätsmedizin Berlin, 13353 Berlin, Germany; ralf.uebelhack@charite.de (R.U.)

**Keywords:** cellobiose, *β*(1→4) glycosidic bond, dose-escalation study, safety, tolerability, fermentable disaccharide, maximum tolerated dose, Bristol Stool Form Scale, Gastrointestinal Symptom Rating Scale, sugar replacer

## Abstract

The disaccharide and innovative ingredient cellobiose, consisting of two *β*-glucose molecules linked by a *β*(1→4) bond is the main component of cellulose. Cellobiose can be used within a wide variety of foodstuffs and functional foods as a low-caloric bulking agent or as a substitute for lactose. For purposes of industrial large-scale production, cellobiose is produced by an enzymatic reaction in which sucrose and glucose are converted to cellobiose and fructose. The goal of this single-arm, dose-escalation study was to evaluate the safety and tolerability of cellobiose and to determine the maximum tolerated dose of cellobiose in healthy subjects. Following a baseline period, consecutive cohorts of six subjects each consumed either single doses of 10, 15, 20 and 25 g, while 12 subjects each received multiple doses of 15 g or 20 g cellobiose (twice daily, 14 days). The main recorded parameters were stool consistency, gastrointestinal well-being (Gastrointestinal Symptom Rating Scale) and adverse events. In each highest single/multiple dosage group, some sensitive subjects experienced flatulence, borborygmus and/or transient diarrhoea. A 100% global tolerability rating makes 20 g cellobiose a tolerable dose for single use. For repeated consumption, we propose up to 15 g cellobiose twice daily (92.6% global tolerability rating). Cellobiose is a promising new ingredient with excellent tolerability.

## 1. Introduction

Among ways to fight obesity and related ailments such as type II diabetes, e.g., weight management programs, sugar reduction in processed foods or the use of medicinal plants with hypoglycaemic effects [1,2] etc. are considered as important options. Since the incidence of type II diabetes is known to be correlated with, for example, a high intake of sugar-sweetened beverages [3], a lower glycemic effect of sweetened drinks (and foods) may be one way of reducing it. However, reducing or replacing sugar in processed foods, especially in solid foods, can be a serious challenge. Thus, an acceptably sweet-tasting, healthy alternative with similar application properties as sucrose, that can be produced large-scale, has immense economic potential.

**Figure 1:**
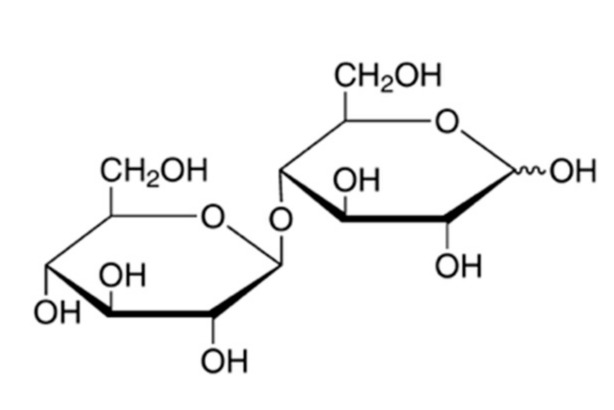
Chemical structure of cellobiose (1-β-d-Glucopyranosyl-4-β-d-glucopyranose).

The disaccharide cellobiose is a naturally occurring reducing sugar consisting of two glucose molecules linked by a beta-1, 4-glycosidic bond (Figure 1). 

In the small intestine of herbivores, cellobiose can be broken down by cellobiosidase and cellulase. However, both enzymes are absent in the human small intestine [4]. Therefore, cellobiose is transported to the colon, where it is fermented by the gut microbiota. End products of this fermentation process are short-chain fatty acids, hydrogen, methane and carbon dioxide [4]; its available energy is about 2 kcal/g (8.4 kJ/g), about half of the energy of sucrose [4], resulting in a lower glycemic and insulinemic effect.

Cellobiose occurs naturally in some foods in small quantities, e.g., in honey [5,6,7], or in fermented or plant-based foods like juices. Industrially, cellobiose can be obtained from the partial hydrolysis of cellulose [8], or by the enzymatic conversion of glucose and sucrose. Cellobiose holds promise as a slightly sweet food ingredient that may be used in certain foods and beverages as a partial or complete sucrose substitute, as a texturizing component or as an energy-reduced bulking agent (Pfeifer & Langen, German Patent DE202016008304U; Givaudan SA, WO2017/068034A1).

Cellobiose was found to be non-mutagenic and non-genotoxic in a bacterial reverse mutation assay and an in vitro micronucleus test using human peripheral blood lymphocytes, respectively. Oral toxicological studies in rats found no adverse effects in a 28 days dose range study or a 90 days repeated dose toxicity study according to OECD408 at doses up to 8.0 g/kg bw/day [9].

Moreover, cellobiose has prebiotic potential and is likely to influence the composition of the intestinal microbiota. In rats, the simultaneous administration of cellobiose and *Lactobacillus rhamnosus* KY-3 over a period of 13 days resulted in an increased number of lactic acid bacteria, especially *L. rhamnosus* in the cecum, whereas the number of Gammaproteobacteria decreased significantly [10].

In an in vitro co-inoculation fermentation model of pig intestines in the presence of *Salmonella,* cellobiose did not reduce the growth of *Salmonella.* However, cellobiose led to high short-chain fatty acid (SCFA) production and a significant increase in the number of *Lactobacillus* spp. [11]. In milk, the addition of cellobiose improved the stability of *Bifidobacterium infantis* [12].

Cellobiose mixed with normal chow was able to reduce dextran sulfate sodium-induced colitis in mice by 9.0% (w/w), indicating that cellobiose may possibly stabilise a beneficial microbial population in the intestines [13].

Additionally, the effects of cellobiose on blood sugar and blood sugar regulation were studied in humans. After ingestion of a single dose of 25 g cellobiose, blood glucose and insulin levels did not increase, whereas these markers increased remarkably with the ingestion of a 25 g glucose control. Cellobiose caused a significantly greater excretion of breath hydrogen gas, indicating microbial fermentation [4].

Furthermore, a three-week double-blind, randomised, placebo-controlled cross-over study investigated the effects of a synbiotic containing *Lactobacillus acidophilus* NCFM (10^9^ CFU) and 5g cellobiose on the composition and metabolic activity of the human gut microbiota in 18 healthy subjects. Self-reported gastrointestinal symptoms did not differ between the investigational product and the placebo group [14]. The synbiotic increased the levels of *Lactobacillus* spp. and relative abundances of the genera *Bifidobacterium, Collinsella*, and *Eubacterium* while the genus *Dialister* was decreased (*p* < 0.05) [11].

Finally, to determine the single dose of cellobiose that causes diarrhoea, a human tolerance study was conducted in Japan. The tested dosages ranged from 15 g to 40 g cellobiose, each dissolved in water and given in the morning between meals. The resulting no-observed-effect level was 0.36 g/kg bw/day, the dosage that induced diarrhoea in 50% of the subjects was 0.612 g/kg bw/day. The highest prevalence in abdominal symptoms after taking cellobiose was observed as “rumbling sound”, “abdominal distension”, and “flatus” in 47%, 39% and 36% of subjects, respectively [15].

Since the broad application of cellobiose in processed foods may result in the continuous intake of substantial doses from various food sources, it is necessary to better understand the tolerability of this sugar. With sets of ascending dosages, the present study aimed at evaluating the tolerable single and multiple doses of cellobiose. Using the default value of 70 kg body weight for adults in the EU [16], a tolerable single dose of 25.2 g cellobiose would result from the above-cited publication [15]. This dose was chosen as a maximum dosage for the single ascending dose (SAD) phase of the current study. The dosages for the multiple ascending dose (MAD) phase were derived from those in the SAD phase.

To our knowledge, this is the first study evaluating the safety of cellobiose in healthy subjects for 14 days.

## 2. Materials and Methods 

### 2.1. Investigational Product

The investigational product (IP) cellobiose (fine white crystalline powder, no further excipients) was obtained from Savanna Ingredients GmbH. Sachets contained either 10 g, 15 g, 20 g or 25 g cellobiose, corresponding to the doses tested in this study. For every intake, a sachet with the appropriate amount was dissolved in a hot herbal infusion and taken together with a meal.

### 2.2. Study Design

The study plan of this single-arm, monocentric, dose-escalation, nutritional study in healthy subjects was examined and accepted by an independent ethics committee (Charité University, Berlin, Germany), and the study was registered under the German Clinical Trials Register (www.germanctr.de) as DRKS00012972. The study was carried out from fall 2017 to spring 2018 at the investigational site analyze & realize GmbH (Berlin, Germany). The study was in conformity with the ethical principles for medical research involving human subjects of the World Medical Association Declaration of Helsinki [17] as well as the EU recommendations for Good Clinical Practice (CPMP/ICH/135/95), ICH E6 (R2).

**Figure 2:**
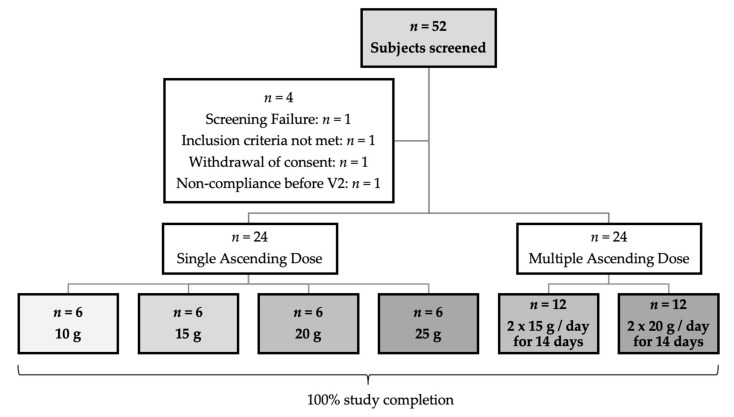
Disposition of study subjects and study flow scheme, showing the actual subject numbers.

**Figure 3:**
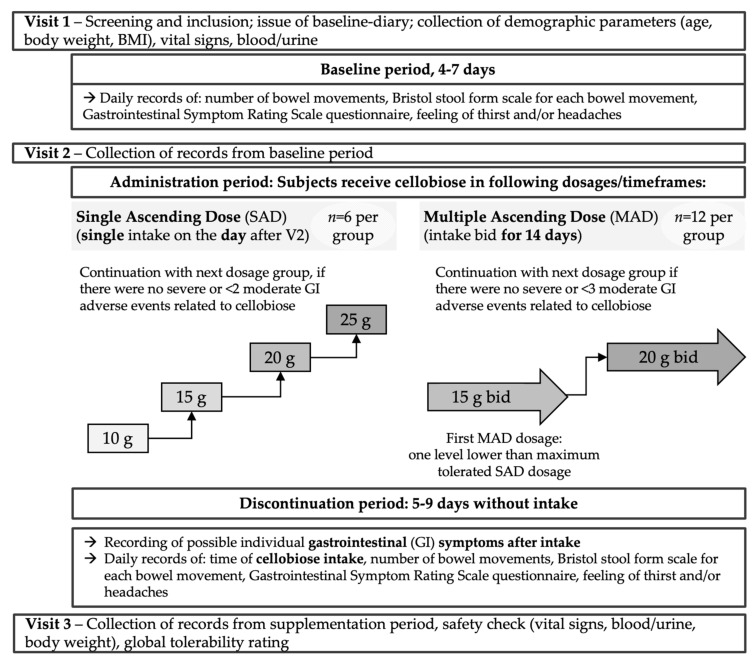
Disposition of study subjects and study flow scheme. SAD phase: single intake of cellobiose 1 day after V2; MAD phase: bid intake of cellobiose for 14 days after V2. Abbreviations: bid, “bis in die” = twice daily; GI, gastrointestinal; SAD/MAD, single/multiple ascending dose; V1, Visit 1 (etc.).

The disposition of study subjects and a study flow scheme are shown in Figure 2 and Figure 3. Following a baseline period, single ascending doses (SAD) were used in 4 dose level cohorts with 6 subjects each: 10 g, 15 g, 20 g and 25 g cellobiose as single dosing in the morning consecutive to Visit V2 (see next paragraph for an explanation of visits). After completion of the SAD phase, two multiple ascending doses (MAD) derived from the results of the SAD phase were used in 2 dose level cohorts with 12 subjects each: first 15 g cellobiose *bid* (twice daily), followed by 20 g *bid*, each for 14 days consecutive to Visit 2, in the morning and afternoon/evening (with at least 8 h in between). 

Each escalating dosage cohort started only after a safety/tolerability evaluation by the principal investigator for the preceding dosage level, and the sponsor’s decision to dose escalate (Figure 3). 

Dose-limiting intolerance within one cohort was defined as follows:

- Moderate adverse event(s) AE(s) with at least probable relationship to IP in the gastrointestinal (GI) tract in 2 subjects (SAD phase) or 3 subjects (MAD phase)
- Severe AE with at least probable relationship to IP in the GI tract in 1 subject (both phases)

The observation of dose-limiting intolerance in the SAD study would result in termination of further dose escalation and the selection of the preceding dose level as Maximum Tolerated Dose (MTD) in the MAD study.

### 2.3. Study Objective and Visits

The study objective was to evaluate the safety and tolerability of cellobiose and to determine the maximum tolerated dose of cellobiose in healthy subjects.

Visits were performed on day 0 (Visit 1 (V1); informed consent, screening, eligibility check, issue of subject diary), after 4–7 days (V2; issue of the IP, return/re-issue of subject diary) and again 5–10 days later within the SAD phase (single-intake-day plus 5–9 days without intake) or 18–23 days later (14 days of intake plus 5–9 days without intake) within the MAD phase (V3; return of used IP and subject diary, safety check (vital signs, blood/urine safety parameters, body weight), global evaluation of IP tolerability). Please see the study flow scheme (Figure 3) for details. 

### 2.4. Recruitment and Inclusion/Exclusion Criteria

Men and women aged 18–65 years, generally in good health without clinically significant findings at the screening visit, with a body mass index (BMI) of 18.5 to 29.9 kg/m^2^, were recruited.

Subjects had to be ready to comply with study procedures, in particular: consumption of the IP as recommended, filling in the subject diary and questionnaire, keeping habitual diet and level of physical exercise and avoiding excessive exercise, avoiding food that contains indigestible or less digestible ingredients such as oligosaccharides or sugar alcohols, avoiding intake of yogurt. Women of childbearing potential had to have negative pregnancy testing (beta HCG-test in urine) as well as commit to use contraception methods during the study. Participation was based upon written informed consent by the participant following written and oral information by the investigator regarding nature, objective, consequences and possible risks of the clinical study. This included that the subjects were informed that they would be ingesting an ingredient that could cause GI symptoms.

Main exclusion criteria were: known allergy or hypersensitivity to the components of the investigational product, history and/or presence of clinically significant self-reported disorder that could influence the conduct and/or outcome, or affect the tolerability of the subject as per the investigator’s judgement, any item of the Gastrointestinal Symptom Rating Scale (GSRS) at screening (1-week recall) scoring >3 or total mean GSRS score >2, relevant deviation of laboratory parameter(s) at screening or any regular medication and/or supplementation that could interfere with the conduct or evaluation of the trial as per the investigator’s judgement.

### 2.5. Assessed Parameters

Body weight (kg) was measured in subjects wearing underwear and no shoes, using calibrated weighing scales (Tanita BC-420MA). 

BMI was calculated as body weight (kg)/(body height (m))^2^. 

The time of cellobiose intake and time (on days one and two days after intake) and the number of bowel movements (BMs) and the respective Bristol Stool Form Scale (BSFS) assessment were recorded in a study diary by each subject. The BSFS defines: separate hard lumps like nuts (hard to pass, type 1); sausage-shaped but lumpy (type 2); like a sausage but with cracks on the surface (type 3); like a sausage or snake, smooth and soft (type 4); soft blobs with clear-cut edges (passed easily, type 5); fluffy pieces with ragged edges, a mushy stool (type 6); watery, no solid pieces, entirely liquid (type 7) [18]. Transient diarrhoea was defined as Type 7 in the study.

In addition, the diary included the GSRS questionnaire, which was filled in daily (24 h recall). The GSRS is a validated questionnaire containing 15 items clustered into 5 dimensions: diarrhoea syndrome (increased passage of stools, loose stools, urgent need for defecation); indigestion syndrome (borborygmus, abdominal distension, eructation, increased flatus); constipation syndrome (decreased passage of stools, hard stools, feeling of incomplete evacuation); abdominal pain syndrome (abdominal pain, sucking sensations, nausea and vomiting); reflux syndrome (heartburn, acid regurgitation) [19,20,21]. For rating of the severity, a seven-graded Likert scale is used (ranging from “no complaints” to “very strong complaints”), resulting in a minimum score of 15 and a theoretical maximum of 105.

Further, the first day of cellobiose intake diary recording comprised questions on the feeling of thirst and/or any headaches (between V1 and V3; 24 h recall), as well as possible individual gastrointestinal (GI) symptoms (vomiting, nausea, discomfort, flatus, distension, borborygmus, tenesmus, upper or lower abdominal pain or other complaints). 

### 2.6. Safety Laboratory Parameters and Vital Signs

At visits V1 and V3, full blood count parameters (haemoglobin, haematocrit, erythrocytes, thrombocytes, and leucocytes) and liver and renal function parameters (alanine transaminase, aspartate aminotransferase, gamma-GT, alkaline phosphatase, bilirubin; creatinine, urea, uric acid) were measured. Additionally, lipid metabolism parameters (total cholesterol, HDL- and LDL-cholesterol), the carbohydrate metabolism parameter glycated haemoglobin HbA1c, glucose and thyroidal parameter TSH were assessed at the screening visit only.

Dipstick urinalyses for the assessment of glucose, proteins and infection parameters were performed at visits V1 and V3. Sitting blood pressure and pulse rate was measured at visits V1 and V3 using standard products and procedures.

### 2.7. Global Evaluation of Tolerability; Dietary Habits/Physical Activity

At visit V3, the subjects and investigators evaluated independently the tolerability of the investigational product by means of a global scaled evaluation with “very good”, “good”, “moderate” and “poor”. At visits V2 and V3, the subjects were questioned with respect to any changes in their dietary habits and physical activity and the respective data was documented.

### 2.8. Statistical Analysis

As the study did not aim at testing a predefined hypothesis, the sample size had been chosen without any statistical consideration. The number of subjects was considered sufficient, acknowledging the interindividual variability of the gut microbiota and physiological processes in the gastrointestinal tract.

Descriptive data analyses were performed, including the calculation of statistical quantities such as arithmetic mean value ± standard deviation (metrically scaled variables), median with interquartile range (ordinally scaled observations and non-normally distributed continuous data) and frequencies (in %) of qualitative variables.

Furthermore, suitable statistical tests were applied to compare groups (exploratory analyses): the nonparametric tests Mann-Whitney U test, Kruskal-Wallis test, Exact F-Test, Wilcoxon test and Log-rank test and the parametric t-test, as appropriate.

## 3. Results

### 3.1. Subjects

Of the 52 screened subjects, four subjects did not receive the investigational product (IP) cellobiose due to the violation of the eligibility criteria (see Figure 2 for details). All of the 48 subjects who received cellobiose completed the study with visit V3. All subjects complied with the foreseen study duration.

The subjects, 30 women and 18 men, had a mean age of 40.5 ± 12.4 years and were all “Caucasian”. There were no statistically significant differences in age, body weight, or body mass index, neither between the dose groups nor between the SAD and MAD groups. 

In the total population, three subjects were affected by anamnestic findings: leg arteries stenosis (SAD group; 15 g), hypertension (one subject in each MAD phase). 12 subjects (12 of 48; 25.0%) took concomitant medication: the two mentioned hypertensive subjects took anti-hypertensive medication; the subject suffering from leg arteries stenosis took acetylsalicylic acid, and nine women from the SAD and MAD phases took oral contraceptives.

A demographic overview and the baseline characteristics of the study population (*n* = 48) are shown in Table 1 and Supplementary Table S1. 

### 3.2. Cellobiose Consumption Related to Body Weight

When the cellobiose intake was related to the subject’s body weights at V2 before start of intake, the dose groups could be classified in daily dose categories ranging from 0.14 to 0.38 g/kg bw/day in the SAD phase and from 0.44 to 0.56 g/kg bw/day in the MAD phase (Table 2).

### 3.3. Body Weight and BMI

There were no relevant changes in body weight and BMI either in the SAD or in the MAD phase (Supplementary Table S2).

### 3.4. Dosage Groups and Maximum Tolerated Dose

The safety/tolerability evaluation of the SAD phase (see Figure 3) established 20 g cellobiose as MTD for single use.

By default, as planned, the MAD phase started with one dosage level lower than the single-use MTD: 15 g *bid*. Since this dosage was well tolerated (according to the predefined escalation rules), the second MAD cohort with 20 g *bid* (*n* = 12) was completed. The safety/tolerability evaluation of the MAD phase (see Figure 3) established 15 g cellobiose *bid* (30 g total per day) as the MTD for repeated consumption.

### 3.5. Individual Gastrointestinal Symptoms

The subjects reported individual gastrointestinal symptoms and their respective timepoint of onset in the diary within the first 24 h after (first) IP intake. The types of individual gastrointestinal symptoms are broadly reflected in the adverse events recorded by the investigators. In the SAD phase, the time to symptom onset ranged between 0.7 and 3 h after intake. In the MAD phase, the time to onset of symptoms ranged between 0.2 and 1.6 h after intake.

### 3.6. Adverse Events

In the SAD phase, AEs rated as possibly/probably related to IP intake occurred in 17% (1 of 6 subjects) with 20 g cellobiose and in 50% (3 of 6 subjects) with 25 g cellobiose (Supplementary Figure S1). In the MAD phase, AEs rated as possibly/probably related to IP intake occurred in 8% (1 of 12 subjects) in the 15 g *bid* group and in 58% (7 of 12 subjects) in the 20 g *bid* group. The AEs possibly/probably related to the IP intake affected mainly the gastrointestinal system, with flatulence, borborygmus and diarrhoea as the most frequent ones (Supplementary Figure S1). There were no serious adverse events.

### 3.7. Frequency of Transient Diarrhoea and Mushy Stools

In all cohorts, the stool consistency by the Bristol Stool Form Scale (BSFS) including the frequency of transient diarrhoea (defined as BSFS type 7) was evaluated, resulting in the following findings: 

Before intake of cellobiose (before V2), three of the total of 274 bowel movements resulted in stools with a BSFS type 6 (mushy stool) (*n* = 1) or 7 (*n* = 2; both from the same person in the MAD group with 15 g *bid*).

In the SAD phase, BSFS type 6 was observed twice in the same subject (on the day of consuming 20 g cellobiose and three days after cellobiose intake). Transient diarrhoea was observed in one subject, two days after consuming 25 g cellobiose.

**Figure 4:**
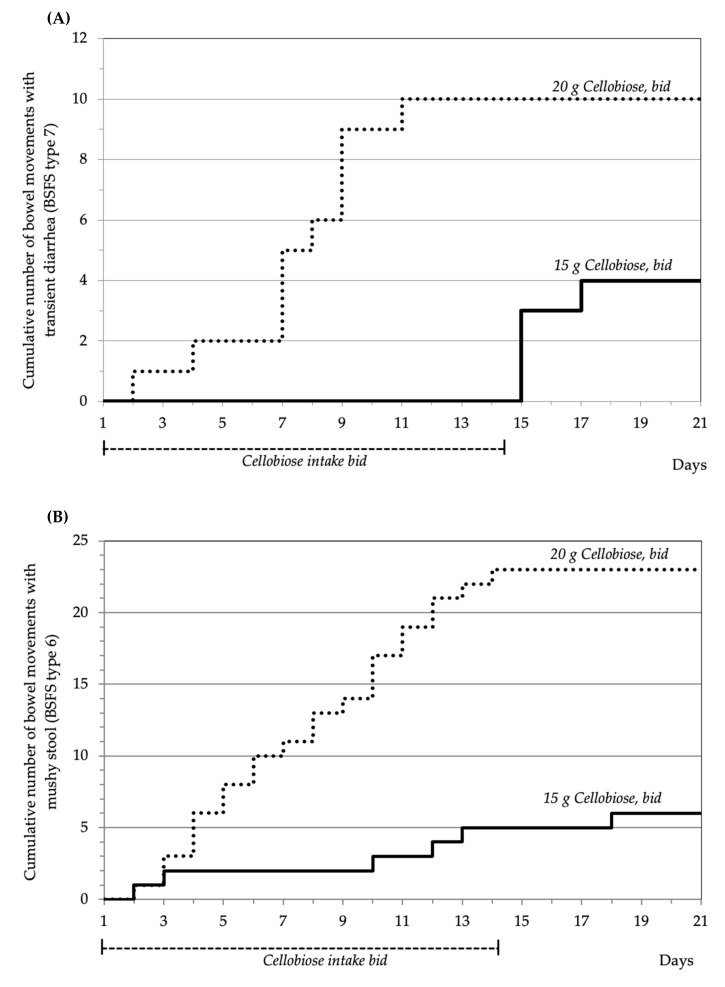
Cumulative bowel movements with transient diarrhoea (BSFS type 7) or mushy stool (BSFS type 6) in the MAD phase. Both MAD groups (*n* = 12 each) are compared, 15 g cellobiose *bid*, versus 20 g cellobiose *bid*. (**A**) Four BSFS type 7 stools in the 15 g cohort were observed in two subjects, ten type 7 stools in the 20 g cohort in four subjects; (**B**) six BSFS type 6 stools in the 15 g cohort were observed in three subjects, 23 type 6 stools in the 20 g cohort in three subjects. Abbreviations: BSFS, Bristol Stool Form Scale; *bid*, “*bis in die*” = twice daily.

In the MAD phase, 15 g cellobiose *bid* were excellently tolerated for the entire intake duration of 14 days, whereas 20 g cellobiose *bid* caused transient diarrhoea during the period of intake (total of 10 stools in four subjects, indicating that some individuals were especially sensitive; Figure 4A). A comparable pattern in the MAD phase groups was observed for the cumulative occurrence of BSFS type 6 (Figure 4B). Interestingly, within the 1–3 days after stopping cellobiose intake, four BSFS type 7 stools (transient diarrhoea) were observed in two subjects in the 15 g *bid* group (Figure 4A).

### 3.8. Time to Bowel Movement

In the SAD phase, there were no relevant differences among the cohorts in the time from intake of cellobiose to the first bowel movement (mean time 7.40 ± 7.79 h).

In the MAD phase, the time to first BM was shorter, with no relevant differences between the dose groups (mean time 2.96 ± 2.65 h for 15 g and 4.46 ± 3.20 h for 20 g).

### 3.9. Bowel Movement Frequency

During the baseline period (4–7 days), on average 0.85 to 1.27 BMs were observed per day (*n* = 48).

In the SAD phase, between V2 and V3, the mean number of bowel movements per day ranged in the 10 g group between 0.50 ± 0.84 and 1.17 ± 0.75, in the 15 g group between 0.67 ± 0.58 and 1.33 ± 0.82, in the 20 g group between 0.50 ± 0.71 and 1.67 ± 0.82 and in the 25 g group between 0.75 ± 0.96 and 4 (four BM were observed in a single subject in this group).

In the MAD phase, between V2 and V3, the mean number of BMs per day ranged in the 15 g *bid* group between 0.67 ± 0.65 and 1.42 ± 0.79 and in the 20 g *bid* group between 0.67 ± 0.65 and 1.33 ± 0.89.

### 3.10. Stool Consistency

There were no relevant differences in the mean type of stool consistency three days before V2 among the cohorts of SAD/MAD phases (see Supplementary Table S1).

The changes in the mean type of stool consistency from three days before V2 (two days after intake) to three days after V2 did not result in statistically significant differences between the cohorts of the SAD phase (*p* = 0.118) or those of the MAD phase (*p* = 0.169). Further, there were no statistically significant differences in the mean type of stool consistency among the dose groups in the SAD phase three days after V2 (*p* = 0.835) or three days before V3 (*p* = 0.684). 

However, there were statistically significant differences in the mean type of stool consistency three days after V2 between the dose groups in the MAD phase (*p* = 0.042), with slightly thinner stools with 20 g cellobiose *bid* (BSFS type 3.9 ± 0.9) versus 15 g cellobiose *bid* (BSFS type 3.1 ± 1.0). Three days before V3 (after stopping the cellobiose consumption), the same comparison did not result in significant differences (15 g *bid* group, BSFS type 3.1 ± 0.8 versus 20 g *bid*-group, BSFS type 3.6 ± 0.8; *p* = 0.207).

### 3.11. Gastrointestinal Well-Being Assessed by the Gastrointestinal Symptom Rating Scale

Between V1 and V2 (baseline period), most of the subjects experienced none or minor GI symptoms. The baseline GSRS score (see Methods section for details regarding the GSRS score) ranged between 15.25 ± 0.68 and 15.96 ± 1.97.

During the SAD phase, very few complaints were reported, which included mostly minor complaints (score 2 of 7 points for most GSRS items). One subject rated one item (“hard stools”) as moderate (3 points) after ingesting 15 g cellobiose. The total GSRS scores ranged in the 10 g group between 15.0 ± 0.0 and 19.5 ± 4.7, in the 15 g group between 17.0 ± 3.2 and 18.7 ± 1.5, in the 20 g group between 15.3 ± 0.5 and 17.0 ± 2.8 and in the 25 g group between 15.0 ± 0.0 and 17.7 ± 2.4.

In the MAD phase, the GSRS rating comprised the entire range from no to very severe complaints. The most affected GSRS items were: rumbling in the stomach, stomach feeling bloated, passing gas or flatus, diarrhoea, loose stools, an urgent need for bowel movement and sensation of incompletely emptying the bowels. The total GSRS score ranged between 15.67 ± 1.61 and 17.67 ± 5.93 for the 15 g *bid* group and between 15.00 and 19.42 ± 7.64 for the 20 g *bid* group.

**Figure 5:**
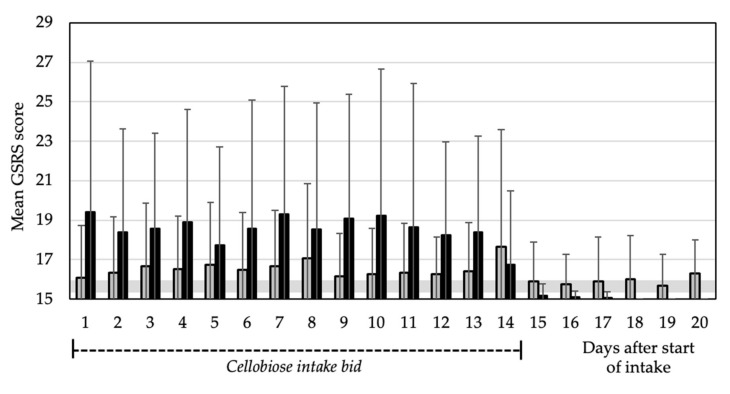
Cumulative Mean Gastrointestinal Symptom Rating Scale (GSRS) score within the MAD groups consecutive to visit V2. Each bar covers a 24-h recall period; the first recall period covers the first day of intake to the second day of intake. 15 g cellobiose *bid*, grey bars; 20 g cellobiose *bid*, black bars. Error bars: standard deviation. The mean baseline GSRS score ranged between 15.25 ± 0.68 and 15.96 ± 1.97. Abbreviations: *bid*, “*bis in die*” = twice daily.

The total GSRS scores for the 20 g *bid* group were higher compared with the total GSRS scores in the baseline period and in the SAD phase. Further, the values in the 20 g *bid* group were higher than in the 15 g *bid* group (Figure 5). After the intake phase, the slightly elevated GSRS values in the 20 g *bid* group returned to baseline level one day after cessation of intake. 

Regarding the GSRS dimensions, the values during the baseline period ranged from 2.0 ± 0.00 to 4.6 ± 1.1, in the SAD phase from 2.0 ± 0.0 to 5.0 ± 1.3 and in the MAD phase from 2.0 ± 0.0 to 6.2 ± 3.3 across the dimensions, with the highest values in the dimensions for indigestion and diarrhoea syndrome (Supplementary Table S3).

The 20 g *bid* group, compared to the 15 g *bid* group, had higher single-item GSRS scores (each *p* < 0.001) for the items that describe rumbling and bloating in the stomach, passing gas or flatus, diarrhoea and loose stools. All other items showed neither group differences nor elevated scores.

### 3.12. Daily Occurrence of Headache and Thirst

In the SAD phase, two subjects were affected by strong thirst, one on the first day (10 g cellobiose) and one on the second day (15 g cellobiose) after intake. Three subjects were affected by headache: One subject on the first two days after IP intake (10 g cellobiose), one subject on the third day after IP intake (15 g cellobiose), and one subject on the second day after IP intake (25 g cellobiose).

In the MAD phase, two subjects were affected by strong thirst on more than one day (both 20 g cellobiose *bid*) after the start of intake. Additionally, three subjects suffered from a headache, two in the 15 g cellobiose *bid* group, one in the 20 g cellobiose *bid* group.

### 3.13. Global Evaluation of Tolerability 

In the SAD phase, the tolerability was rated “good” or “very good” by subjects and investigators in 100% of cases in each 10 g, 15 g and 20 g group, and in 50% of cases in the 25 g group. In the MAD phase, the tolerability was rated “good” or “very good” by subjects and investigators in 92.6% of cases in the 15 g *bid* group and in 66.7% of cases in the 20 g *bid* group.

### 3.14. Safety Laboratory Parameters and Vital Signs 

There were no relevant differences among the cohorts with respect to safety laboratory parameters and vital signs at the screening. Following cellobiose consumption, there were no clinically relevant changes in the safety parameters.

## 4. Discussion

In the present study, cellobiose was dissolved in a herbal infusion and administered together with a meal, to simulate real-life consumption as a food ingredient. As seen from this study, and as expected, the quantity of cellobiose that is tolerated without adverse events is limited. This is plausible, since microbial fermentation of cellobiose is bound to result in the production of gaseous fermentation products; also, high amounts of cellobiose may cause diarrhoea. On the other hand, microbiota-generated SCFA are known for their role in providing extra energy to the host, as well as regulating energy homeostasis, thereby counteracting metabolism disorders and its associated diseases such as obesity and type 2 diabetes [22]. Accordingly, providing cellobiose as a substrate for potential SCFA generation [14] may be advantageous to health [14].

In vitro studies have shown that the addition of cellobiose to a culture inoculated with human faeces increased bifidobacteria numbers and butyric acid concentrations compared to a control or a culture to which fructooligosaccharides were added instead. The authors calculated a higher prebiotic index for cellobiose, compared to the control or fructooligosaccharides [23]. Accordingly, it was hypothesized that cellobiose may be more tolerable to people with intolerance towards FODMAPs, fermentable oligo, di- and monosaccharides (such as lactose or fructose), who suffer from digestive discomfort such as gas, pain and bloating, sometimes coupled with diarrhoea. Clinical data remains to be generated.

Stool consistency was assessed via the Bristol Stool Form Scale questionnaire [18]. The BSFS has been widely used in research and clinical settings and recommended by both the Rome Foundation for functional gastrointestinal disorders [24] and the United States’ Food and Drug Administration [25]. It has been shown to correlate to gut transit time [26,27], as well as to stool hardness and dry matter [28]. Moreover, substantial validity and reliability have been demonstrated for the BSFS [29]. Similarly, the GSRS is a validated questionnaire, also in the German language [30]. As the tools applied in the present study have been appropriate and reliable, the results obtained may be considered robust. 

When administered as a single dose, adverse events like flatulence plus borborygmus (*n* = 2) and diarrhoea (*n* = 1) occurred in 50% of the subjects (3 of 6) after consuming 25 g cellobiose, whereas 20 g cellobiose resulted in only one case of flatulence plus borborygmus among six subjects (17%). No adverse GI events were caused by 10 g or 15 g cellobiose. There were no significant stool consistency changes (three days after V2 compared to baseline) when comparing the cohorts of the SAD phase. In one case, diarrhoea occurred after consuming 25 g cellobiose, this normalised again one day later. In all single-dose groups, the mean GSRS scores remained in the same range (15.0 ± 0 and 19.5 ± 4.7). Based on the observed low level of adverse events, BSFS types and the GSRS results, as well as on the 100% global tolerability rating, the tolerable cellobiose intake level was set to 20 g cellobiose daily for single use. Higher acute doses may result in a substantial level of adverse GI events, as observed in this study with 25 g cellobiose. This dose approximately corresponds to the literature data with a single dose no-observed-effect level of 0.36 g/kg bw/day [15] (25.2 g for a 70 kg person) [15]. 

There were clear differences between the two multiple-dose groups (20 g *bid* versus 15 g cellobiose *bid*): Whereas 20 g cellobiose *bid* caused a GI adverse event rate of 58%, the GI AE rate was 8% in the 15 g *bid* group. The mean stool consistency was significantly thinner in the 20 g cellobiose *bid* group compared to the 15 g *bid* group three days after V2; also, in the 20 g *bid* group (but not in the 15 g *bid* group), the GSRS values were elevated during intake (Figure 5). These results, as well as the 92.6% global tolerability rating for the 15 g *bid* group, support a tolerable dose of repeated cellobiose consumption of 15 g twice daily (30 g total). This daily dose is 6 × higher than what was previously reported as the multiple-dose in literature [14].

Some parameters did not result in any group differences, e.g., time of bowel movement, bowel movement frequency, occurrence of headache/thirst or safety laboratory parameters and vital signs. Considering that the upper SAD and MAD dosage groups only had slightly more loose stools, this is plausible. Increased bowel movement frequencies would only be expected for more severe diarrhoea. To confirm safety, it is reassuring that laboratory safety parameters did not change in a clinically relevant manner. The occurrence of headache or thirst did not correlate with the dosages and may therefore not be related to cellobiose intake.

As to be expected, some individuals were more sensitive to cellobiose than others, regarding gastrointestinal disturbances. For example, the two subjects that suffered from the adverse event recurrent diarrhoea after the intake of 20 g cellobiose *bid* also suffered from flatulence. At the same time, five of 12 individuals did not experience any GI events at this high dose.

Additionally, some of the recorded GI events may have been unspecific and not related to the cellobiose intake. In this regard, one subject that ingested 15 g cellobiose *bid* experienced diarrhoea three days after cellobiose discontinuation (Figure 4A). However, the same subject also experienced diarrhoea on day five of the baseline period; accordingly, the observed diarrhoea on day 17 may have been a result of a general tendency towards GI disturbance, rather than a (late) cellobiose effect.

There are limitations to this study, mainly the small number of subjects per group, and the rather short duration of intake (14 days) in the multiple dosing groups. Additionally, a placebo control was not included; however, baseline data can—to some extent—substitute the placebo data, considering that this is (only) a safety study. In spite of the limitations, the available data clearly show gastrointestinal tolerability up to the suggested maximum tolerable dose levels. A future randomised controlled study over an extended time period is recommended to strengthen the present data.

## 5. Conclusions

As a conclusion, this first study evaluating cellobiose in healthy subjects for 14 days provides evidence of gastrointestinal tolerance at lower dosages (up to 15 g twice daily or 20 g once daily) and a fairly good safety profile at the highest dosage tested (25 g daily or 20 g twice daily). Mild signs of intolerance expressed as gastrointestinal complaints were observed in sensitive subjects, mostly confined to flatulence and transient diarrhoea. Based on the study results, the tolerable dose of cellobiose consumption is proposed to be 20 g cellobiose daily for single-use and 15 g twice daily (30 g total) for repeated consumption.

## Figures and Tables

**Table 1:** Baseline characteristics—gender, age, body weight, BMI.

	Dosage of Cellobiose	*n*	Gender (f/m)	Age (years)		Body Weight (kg)		BMI (kg/m^2^)	
				**Mean**	**SD**	**Mean**	**SD**	**Mean**	**SD**
Total	--	48	30/18	40.5	12.4	72.57	12.42	24.17	2.90
**SAD**	--	**24**	**13/11**	**43.0**	**12.5**	**72.61**	**12.46**	**24.00**	**2.49**
Dose 1	10 g	6	3/3	40.5 ^a^	12.8	70.90 ^b^	10.95	24.30 ^c^	2.94
Dose 2	15 g	6	3/3	50.8 ^a^	12.1	75.28 ^b^	16.02	24.63 ^c^	3.50
Dose 3	20 g	6	3/3	42.7 ^a^	13.0	78.18 ^b^	13.15	24.24 ^c^	1.91
Dose 4	25 g	6	4/2	38.2 ^a^	11.5	66.07 ^b^	7.98	22.83 ^c^	1.31
**MAD**	--	**24**	**17/7**	**38.0**	**12.0**	**72.52**	**12.65**	**24.35**	**3.31**
Dose 1	15 g *bid*	12	10/2	38.1 ^a^	11.2	70.00 ^b^	9.57	23.41 ^c^	2.34
Dose 2	20 g *bid*	12	7/5	41.0 ^a^	12.6	75.05 ^b^	15.13	25.28 ^c^	3.93
***p* value (SAD vs. MAD)**		--	--	**0.176**	--	**0.996**	--	**0.721**	--

^a^*p* = 0.263 for SAD Doses 1-4; *p* = 0.259 for MAD Doses; ^b^ *p* = 0.407 for SAD Doses 1-4; *p* = 0.370 for MAD Doses; ^c^ *p* = 0.619 for SAD Doses 1-4; *p* = 0.178 for MAD Doses. Abbreviations: BMI, body mass index; BSFS, f/m, female/male; SD, standard deviation; *bid*, “*bis in die*” = twice daily; SAD/MAD, single/multiple ascending dose.

**Table 2:** Cellobiose consumption related to body weight.

Phase	Dosage	Range (g/kg bw/day)	Mean (g/kg bw/day)	SD
SAD phase	10 g/day	0.12–0.19	0.14	0.02
	15 g/day	0.15–0.28	0.21	0.04
	20 g/day	0.22–0.35	0.26	0.05
	25 g/day	0.34–0.44	0.38	0.04
MAD phase	15 g *bid* (30 g/day)	0.35–0.54	0.44	0.06
	20 g *bid* (40 g/day)	0.42–0.82	0.56	0.12

Abbreviations: bw, body weight; *bid*, “*bis in die*” = twice daily; SAD/MAD, single/multiple ascending dose; SD, standard deviation.

## Supplementary Materials

The following are available online at https://www.mdpi.com/2072-6643/12/1/64/s1, Table S1: Baseline characteristics—Bowel movements/day, BSFS types; Table S2: Changes in body weight and BMI; Table S3: GSRS dimensions during the study phases; Figure S1: Gastrointestinal adverse events after first intake with probable or possible connection to cellobiose intake.
