# Supplementary material for: A Dose-Escalation Study Demonstrates the Safety and Tolerability of Cellobiose in Healthy Subjects
Source: Nutrients. 2019 Dec 25;12(1):64. doi: 10.3390/nu12010064 (PMC7019479; doi:10.3390/nu12010064)
Supplement: Supplementary file 1 [file nutrients-12-00064-s001.pdf]

nutrients

Supplementary Material for the Article

A Dose-Escalation Study Demonstrates the Safety and Tolerability of Cellobiose in Healthy Subjects

Margret Irmgard Moré [1], Elisa Postrach [1,*], Gordana Bothe [1], Sonja Heinritz [2] and Ralf Uebelhack [3]

[1] analyze & realize GmbH, 13467 Berlin, Germany; mmore@a-r.com (M.I.M.); gbothe@a-r.com (G.B.)
[2] Savanna Ingredients GmbH, 27404 Elsdorf, Germany; sonja.heinritz@savanna-ingredients.com
[3] Charité - Universitätsmedizin Berlin, 13353 Berlin, Germany; ralf.uebelhack@charite.de
* Correspondence: epostrach@a-r.com (E.P.); Tel.: +49-30-4000-8130

Table S1. Baseline characteristics – bowel movements/day, BSFS types

	n	Mean number of BM per day	SD	Subjects with no BM [%]	Stools with BSFS type [%]			
					type 1 [a]	type 2-5 [a]	type 6 [a]	type 7 [a]
Day 1	48	1.06	0.52	10.4	4.2	85.4	0	0
Day 2	48	1.00	0.46	18.8	4.2	77.1	**0**	**0**
Day 3	48	1.04	0.58	18.8	4.2	77.1	0	0
Day 4	48	1.02	0.60	20.8	2.1	77.1	0	0
Day 5	43	0.85	0.68	23.3	7.0	65.1	2.3 [b]	2.3 [b]
Day 6	25	0.88	0.60	24.0	0	76.0	0	0
Day 7	11	1.27	0.65	9.1	0	90.9	**0**	**0**

[a] of the first bowel movement of the day. Depending on the day, 12.0%-36.4% of the subjects had a second bowel movement; 0%-8.7% of the subjects had a third bowel movement, and a fourth bowel movement was recorded once (on day 4, type 2). Within the 2nd-4th bowel movements, there was none with BSFS 6 and one with BSFS 7 (MAD group 15 g; third bowel movement of the day; the second bowel movement for this subject was of type 1). [b] From MAD group 15 g cellobiose *bid*; Abbreviations: *bid*, "*bis in die*" = twice daily; BMI, body mass index; BM, bowel movement(s); BSFS, Bristol stool form scale; SD, standard deviation; SAD/MAD, single/multiple ascending dose.

Table 2. Changes in body weight and BMI.

**Change in body weight [kg] v3-v2**	***n***	**Mean**	**SD**	**Min**	**Median**	**Max**
SAD 10 g	6	-0.05	0.29	-0.4	-0.05	0.3
SAD 15 g	6	-0.02	0.38	-0.3	-0.20	0.6
SAD 20 g	6	-0.08	0.40	-0.7	-0.10	0.4
SAD 25 g	6	-0.05	0.26	-0.3	-0.15	0.4
MAD 15 g *bid*	12	0.24	0.37	-0.2	0.15	1.0
MAD 20 g *bid*	12	-0.17	0.56	-1.2	-0.20	0.7
**Change in BMI [kg/m²] v3-v2**	**n**	**Mean**	**SD**	**Min**	**Median**	**Max**
SAD 10 g	6	-0.02	0.10	-0.14	-0.02	0.10
SAD 15 g	6	-0.01	0.12	-0.10	-0.07	0.18
SAD 20 g	6	-0.02	0.12	-0.18	-0.04	0.15
SAD 25 g	6	-0.02	0.09	-0.09	-0.05	0.15
MAD 15 g *bid*	12	0.08	0.12	-0.06	0.05	0.33
MAD 20 g *bid*	12	-0.07	0.18	-0.42	-0.07	0.21

Abbreviations: *bid*, "*bis in die*" = twice daily; BMI, body mass index; SAD/MAD, single/multiple ascending dose.

Table S3. GSRS dimensions during the study phases

**GSRS item**	**Range- Baseline [a]**				**Range- SAD [b]**				**Range- MAD [b]**			
**(number of items)**	**LM**	**SD**	**HM**	**SD**	**LM**	**SD**	**HM**	**SD**	**LM**	**SD**	**HM**	**SD**
Abdominal pain (3)	3.05	0.22	3.17	0.48	3.00	0.00	3.46	0.83	3.00	0.00	3.17	0.82
Reflux syndrome (2)	2.00	0.00	2.05	0.22	2.00	0.00	2.12	0.45	2.00	0.00	2.04	0.42
Diarrhoea syndrome (3)	3.00	0.00	3.08	0.35	3.00	0.00	3.54	1.47	3.00	0.00	3.61	2.71
Indigestion syndrome (4)	4.12	0.45	4.56	1.13	4.00	0.00	5.04	1.27	4.29	1.00	6.17	3.28
Constipation syndrome (3)	3.00	0.00	3.27	0.79	3.00	0.00	3.46	1.06	3.04	0.20	3.38	1.35

[a] day-1-7 of baseline period; [b] all days consecutive to visit V2. Abbreviations: GSRS, Gastrointestinal Symptom Rating Scale; SD, standard deviation; SAD/MAD, single/multiple ascending dose; LM, lowest mean, HM, highest mean.

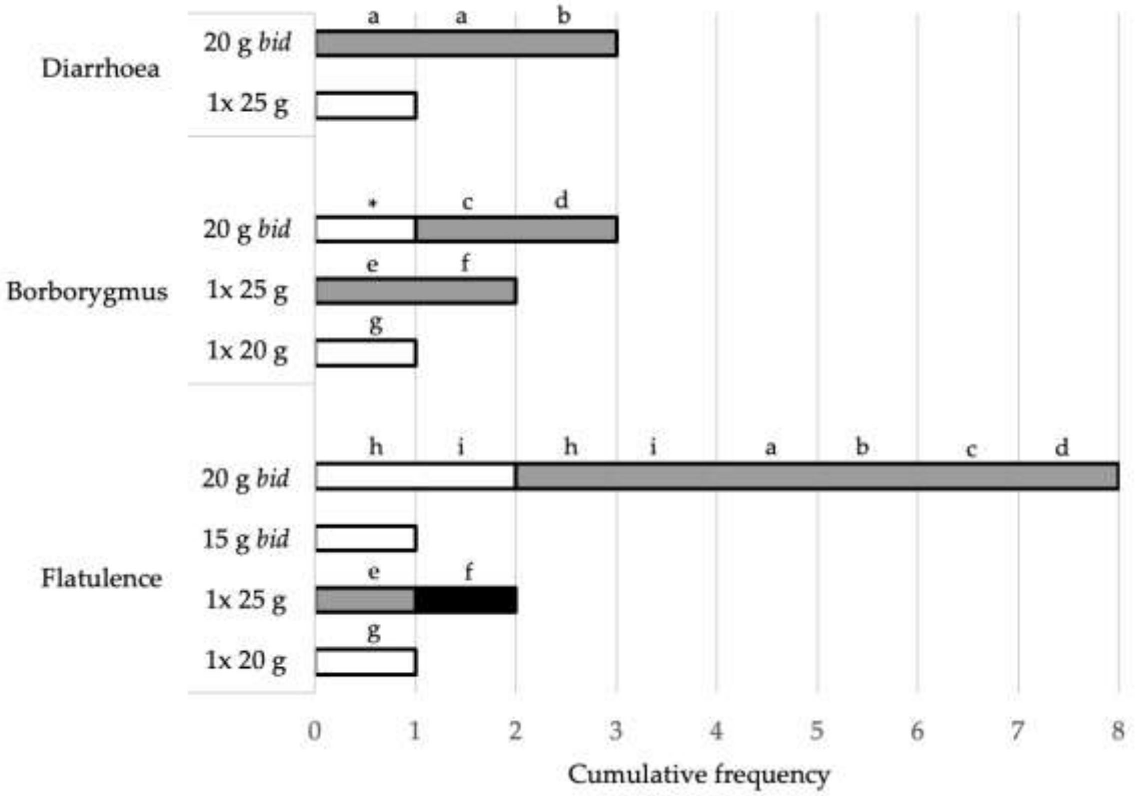

Figure 1. Gastrointestinal adverse events after first intake with probable or possible connection to cellobiose intake. This figure shows the number of the adverse events with probable or possible connection to cellobiose intake (diarrhoea, borborygmus, flatulence) by cohorts. Shading stands for light (white), moderate (grey) or severe (black) adverse events. The dosage of cellobiose is indicated. Dosage groups without AEs are not shown. Small letters indicate identical subjects with multiple adverse events. The subject marked by an asterisk also suffered from “light discomfort”. Abbreviations: *bid*, “*bis in die*” = twice daily.
